# FoxO3a suppresses neuropeptide W expression in neuronal cells and in rat hypothalamus and its implication in hypothalamic-pituitary-adrenal (HPA) axis

**DOI:** 10.7150/ijbs.45619

**Published:** 2020-08-25

**Authors:** Fengxia Yan, Rikang Wang, Shuai Li, Xia Zhao, Yizhou Jiang, Linlin Liu, Jiankang Fang, Xuechu Zhen, Philip Lazarovici, Wenhua Zheng

**Affiliations:** 1 Center of Reproduction, Development & Aging and Institute of Translation Medicine, Faculty of Health Sciences, University of Macau, Macau, China; 2 School of Medical Science, Jinan University, Guangzhou, China; 3 National Pharmaceutical Engineering Center for Solid Preparation in Chinese Herbal Medicine, Jiangxi University of Traditional Chinese Medicine, Nanchang 330006, P. R. China; 4 Jiangsu Key Laboratory of Neuropsychiatric Diseases and College of Pharmaceutical Sciences, Soochow University, Suzhou, Jiangsu 215123, China; 5 School of Pharmacy Institute for Drug Research, Faculty of Medicine, The Hebrew University of Jerusalem, Jerusalem 91120, Israel

**Keywords:** Glucocorticoids, Neuropeptide W (NPW), FoxO3a, HPA axis dysfunction, Hypothalamus

## Abstract

FoxO3a, a forkhead family member of transcription factors, is involved in the regulation of cell metabolism, proliferation, differentiation and apoptosis. However, whether FoxO3a participates in the regulation of glucocorticoids induced-hypothalamic-pituitary-adrenal (HPA) dysfunction is still unknown. Our present results indicate that dexamethasone(DEX) increased FoxO3a expression in PC12 and hypothalamic neuronal cultures in correlation to reduced expression of NPW, a process that could be blocked by GR2 antagonist. DEX restrained the phosphorylation of Akt and FoxO3a, but not ERK1/2 phosphorylation, resulting with FoxO3a nuclear localization. Overexpression of FoxO3a inhibited NPW expression, while FoxO3a knockdown by siRNA had the opposite effect. The regulatory region of NPW promoter contains multiple FoxO3a binding sites, and FoxO3a bonding to these sites inhibited its transcriptional activity. In a rat model, chronic administration of corticosterone reduced animals' body weight and sucrose consumption and caused stress- depression like behavior. Corticosterone treatment induced a marked increase in FoxO3a levels, while decreased the expression of NPW protein in the hypothalamus. Immunofluorescent double labeling demonstrated that FoxO3a and NPW were collocated in the hypothalamus. Taken together, these data indicate that NPW is a new direct downstream target gene of FoxO3a. FoxO3a suppressed the transcription of NPW and modulated glucocorticoids-induced HPA dysfunction by directly regulating the expression of NPW. Thus, present findings suggest that FoxO3a and NPW may be potential therapeutic targets for endocrine and psychiatric disorders.

## Introduction

Glucocorticoids (GC) exert widespread actions in central nervous system, affecting gene transcription, synaptic activity and behavior. While GC mainly act to maintain homeostasis by inducing physiological and behavioral adaptation, prolonged exposure to elevated GC levels may result in neurological and psychiatric disorders 1. Natural and synthetic GC act on the hypothalamus and pituitary gland to suppress hypothalamic-pituitary-adrenal (HPA) activity, representing an important endocrine feedback regulation mechanism 2. In recent years, growing evidence suggests that a number of neuropeptides, including Neuropeptide W (NPW), are also important regulators of the HPA axis 3. Neuropeptide W (NPW) is a peptide hormone, originally isolated from porcine hypothalamus, that plays an important role in the regulation of feeding and drinking behavior, stress responses, emotion, anxiety and fear 4-6. It represents an endogenous ligand for the orphan G protein-coupled receptors NPBWR1 (GPR7) and NPBWR2 (GPR8) 7. Two endogenous molecular isoforms of NPW ligands that consist of 23- and 30-amino acid residues and bind with similar affinity to their receptors were reported 7, 8. The NPW and their receptors are highly expressed in several discrete regions of the rodent brain, including the hypothalamic paraventricular nucleus (PVN), arcuate nucleus (ARC), ventromedial nucleus (VMH) and lateral hypothalamus (LH) 9, 10. The fact that brain administered NPW caused a dose-dependent increase in corticosterone (CORT) levels in rats indicates that NPW may play an important role in the hypothalamic organization of the endocrine response to stress and the modulation of the HPA axis 11.

FoxO3a (FKHRL1) is a member of a subgroup of the Fork head family of transcription factors, characterized by a conserved DNA-binding domain (the 'Fork head box', or FOX), involved in cell growth, survival, metabolism and longevity, as well as stress responses and learning activity 12-18. Akt is a serine/threonine-specific protein kinase that plays a key role in multiple cellular processes like glucose metabolism, apoptosis, cell proliferation, transcription and cell migration 19-21. Although multiple kinases are able to phosphorylate various residues in the FoxO3a proteins, growth factors mainly regulate the functions of FoxO3a by activating the phosphoinositide 3-kinase (PI3K)/Akt/serum-and glucocorticoid-regulated kinase (SGK) (PI3K/Akt/SGK) axis 19, 21, 22. In the absence of growth factors stimulation, FoxO3a proteins are in a non-phosphorylated form and localized in the nucleus where they bind to the promoter of their respective gene targets, regulating transcription, apoptosis, and oxidative stress 23. By contrast, activation of the PI3K/Akt/SGK pathway induced by growth factors, causes FoxO3a phosphorylation leading to the translocation of FoxO3a from the cell nucleus to the cytoplasm, affecting cell cycle regulation, scavenging of ROS and control of protein homeostasis 19, 20, 22. FoxO3a can mediate the expression level the palmitoyl transferase zDHHC3, which plays important role in hippocampal synaptic plasticity and memory 24. Downregulation of FoxO3a conferred neuroprotective effects by inhibiting the autophagy in the neurons 25. In addition, the activation of FoxO3a was correlated to the appearance of depression, but FoxO3a knockout significantly reduced depression-like behavior in transgenic animals 17. The specific distribution of FoxO3a in the brain and these different neuroscience findings support the hypothesis that FoxO3a may have important functions in the central nervous system.

We have previously found that GC induced FoxO3a nuclear translocation in correlation to inhibition of the PI3K/Akt signaling pathway in PC12 neuronal cell cultures 26. In addition, GC have been also shown to down-regulate NPW expression in hypothalamus 27. We thus hypothesized that GC could activate FoxO3a in the hypothalamus, which in turn may mediate the suppression of neuropeptide W protein expression, representing a signaling mechanism contributing to the regulation of HPA axis/ GC -feedback mechanism. To investigate this hypothesis, we treated PC12 cell with and without overexpression of FoxO3a and hypothalamic neuron with the typical synthetic GC, dexamethasone, and rats with natural GC, corticosterone, and measured FoxO3a phosphorylation and expression in correlation with NPW protein and mRNA expression levels. We found that GC significantly increased FoxO3a levels while decreased the expression of NPW in neuronal cultures and in rat's hypothalamus in a time and dose dependent manner. This indirect correlation supports our hypothesis that NPW represents a novel pivot target of FoxO3a in the regulation of the HPA axis.

## Materials and Methods

### Materials

Dexamethasone (DEX, CAS Number: 50-02-2), Corticosterone 21-acetate (CORT, 98%; CAS Number: 1173-26-8), were purchased from Sigma-Aldrich (USA). Human recombinant NGF was obtained from Upstate Biotechnology (Lake Placid, NY, USA). Glucocorticoid receptor inhibitor RU-486 was purchased from Calbiochem (La Jolla, CA, USA). Specific antibodies towards p-FoxO3a, p-AKT, T-AKT, p-ERK1/2, T-ERK1/2, GAPDH and β-actin were obtained from Santa Cruz Biotechnology (Delaware Ave, Santa Cruz, USA). Specific antibody towards NPW was obtained from Biobyt (Wuhan, China). Antibody against FoxO3a was obtained from Cell Signaling Technology (Woburn, USA). Antibody against CRH was obtained from Signalway antibody (Pearland, Texas, USA). GFP-N1 and GFP-FoxO3a were kindly provided by Dr. Marten P. Smidt, University Medical Center, Utrecht, Holland. The primers of RPL-19, FoxO3a and NPW were obtained from Invitrogen Co. Ltd. (Guangzhou, China). FoxO3a-siRNA was purchased from GenPharma Co. Ltd. (Shanghai, China).

### Cell cultures

PC12 cells were originally provided by Dr. Gordon Guroff (National Institute of Child Health and Human Development, National Institutes of Health, Bethesda, MD, USA) and cultured as described by Zheng et al 20. PC12 cells were cultured in DMEM medium supplemented with 10% fetal bovine serum (FBS), 100 µg/ml streptomycin/100 units/ml penicillin and incubated at 37℃ in a 5% CO2 humidified atmosphere. PC12 cell cultures were weekly splitted at 1:5 ratio. Primary hypothalamic neuronal cultures were prepared from fetal rat brains obtained on embryonic day 18, as described 28, and cultured for 7-8 days in DMEM/F12 supplemented with 2% B 27, 20 μM L-glutamine, 15 mM HEPES, 10 U/ml penicillin, and 10 μg/ml streptomycin.

### Animals

Thirty Male Sprague-Dawley rats weighting 140-160 g were purchased from the Laboratory Animal Center of Sun Yat-sen University. All animal procedures confirmed with the China Animal Welfare Legislation were reviewed and approved by the Sun Yat-sen University Committee on Ethics in the Care and Use of Laboratory Animals. (Animal quality certificate No.: 0005201). The protocol of corticosterone (CORT) administration was performed as previous reports 29, 30. Rats were minimally handled (about 1 min) for seven consecutive days, prior to CORT treatment. Animals were randomly assigned to model group (n=15), which were given subcutaneous injection of corticosterone 21-acetate (CORT, 40 mg//kg), and normal control group (n=15), which were injected with equal volume of saline solution (Vehicle). Treatment and saline administrations were performed subcutaneously once per day, for 21 consecutive days between 9-11 am. The rat body weight was weekly measured and sucrose consumption carefully tested. After the beginning of the experiment, on day 22, each rat was tested with forced swim test, a commonly used rodent behavioral assay aimed to evaluate antidepressant drugs efficacy and experimental manipulations that are aimed at rendering or preventing depressive-like situations.

### RT-PCR assay

Total RNA was extracted from PC12 cells with TRIzol (Invitrogen, Carlsbad, CA, USA) according to the manufacturer's instructions. Real time PCR was performed using All-in-One First-Strand cDNA Synthesis Kit (Tiangen, Beijing, China) following the manufacturer's protocol. The amplification number of cycles was 34, and the reaction took place for 5 min at 94 °C and 94 °C for 30 s, annealing at 54 °C for 30 s, then extension at 72 °C for 1 min, and extension at 72 °C for 5 min. The predicted 380-bp PCR product of NPW was amplified.

NPW primer sequences were: 5'-ACTGCTGCTTCTGCTCTTGC-3' (sense) and 5'-GCGTCTCACCGAAGGCTCTA-3' (anti-sense). FoxO3a primer sequences were: 5′-GGAACGUGAUGCUUCGCAATT-3′ (sense) and 5′-UUGCGAAGCAUCACGUUCCTT-3′ (anti-sense). RPL-19 expression was chosen as an internal control. RPL-19 primer sequences were: 5′-ATCGCCAATGCCAACTCT-3′ (sense) and 5′-GAGAATCCGCTTGTTTTTGAA-3′ (anti-sense).

The PCR products were detected with ethidium bromide staining by 1.2 % agarose gels. Results were normalized with those obtained from RPL-19.

### Western blotting assay

Western blotting assay was performed as described previously 21. Briefly, the cells from different experimental conditions were collected and lysed in RIPA buffer. Samples with equal amounts of protein were separated using 8% SDS-PAGE and transferred to PVDF membrane. Then, the membrane was blocked with 5% non-fat milk in TBST for 1-2 h at room temperature. The primary antibodies against NPW, FoxO3a, p-FoxO3a, p-AKT, T-AKT, p-ERK1/2, T-ERK1/2, β-actin and GAPDH were used for detecting respective proteins. The membranes were then washed with TBST and incubated with secondary antibody conjugated with HRP. The protein bands were detected using chemiluminescence. Bands intensity was analyzed using image J analysis software.

### FoxO3a translocation studies in PC12 cells

PC 12 cells were applied to 12-well plates containing coverslips and grown in DMEM medium containing with 10 % FBS and 1% penicillin/streptomycin, and were maintained at 37 °C in a humidified, 5% CO2 atmosphere. The next day, the cells grown on coverslips were transfected for 6 h with 1 μg GFP-FoxO3a plasmid using Lipofectamine 2000. Then, media was replaced with DMEM plus 10% serum for 24 h. In the study of translocation, the culture medium was replaced with DMEM plus 10 % serum and treated with 1 μM DEX or co-treated with 1 µM DEX and 1µM RU486 for 60 min. Then the cells were fixed in 4% PFA for 10 min at room temperature. Fixed cells were then washed with 1xPBS for three times. The coverslips were then mounted on glass slides using one drop of antifade mounting medium with DAPI (P0131, Beyotime). The images were acquired using a Nikon A1 confocal microscope.

### DNA chip assay

Chromatin immunoprecipitation (ChIP) assay was used to detect the localization of DNA binding sites of FoxO3a on the promoter region of NPW. In brief, hemagglutinin-FoxO3a was transfected into PC12 cells to prepare soluble chromatin. Then, the soluble chromatin was adjusted to a proper concentration with chromatin immunoprecipitation-dilution buffer and pre-cleared with protein A/G beads. The precleared chromatin solution was thereafter used for immunoprecipitation assay with either anti-FoxO3a, anti-β-actin or anti-IgG antibodies. Following washing, the antibody-protein-DNA complex was eluted from the beads by re-suspending the pellets in 1% SDS and 0.1 M NaHCO3 at room temperature for 20 min. After reversible cross-linking, protein and RNA were removed by incubating the solution with proteinase K and RNase A for 3 h at 42 °C. Purified DNA was subjected to PCR with primers specific for the putative FoxO3a-binding sites in the NPW promoter described in JASPAR database (http://jaspar.genereg.net/). The primers for PCR were as follow: 5′-TCTACCGCTGAGCTAAATCC-3′ (sense); 5′-CGTCCCTCTTCCTGCCTA-3′ (antisense) and 5′ATGCTTAAAACTTTCCTGTC (sense) and AGCCTCCCAATGAGTAGAT3′ (antisense), respectively.

### Dual-Luciferase reporter assay

We defined a-2-kb sequence from the transcription starting site using bioinformatics software tools (http://genome.ucsc.edu/) and searched for the binding sites of FoxO3a. To study whether FoxO3a regulates the promoter of NPW, PC12 cells were applied to 48-well plates and transiently transfected by Lipofectamine 2000 approach using either pGL3-NPW-FoxO3a for reporter plasmid or pGL3-TK plasmid for Renilla luciferase-encoding plasmid. After transfection for 36 h, the cells were collected, lysed and centrifuged for 1 min at 12, 000 rpm, then luciferase expression was measured using Dual-Luciferase ® Reporter Assay System (Promega Co. Medison,WI, USA Cat Number E1910).

### Immunohistochemistry and Immunofluorescence

Hypothalamic neurons were processed for immunohistochemically staining as previously described 10. Rats were first perfused transcardially with 0.1 M phosphate buffer (pH 7.4), then with 4% paraformaldehyde in 0.1 M phosphate buffer. The hypothalamus was sectioned into 40 μm slices at -20 °C using a cryostat. Hypothalamic sections were incubated for 2 days at 4℃ with primary antibodies anti-FoxO3a (1:100, #12829, Cell Signaling), then were stained with the avidin-biotin method. Using a double staining similar procedure, co-localization of CRH with FoxO3a and NPW with FoxO3a were also estimated. Briefly, the neurons were fixed with 4% paraformaldehyde for 5 min at room temperature and permeated with 0.5% Triton X-100 for 30 min. The neurons were then blocked with 3% horse serum (Sigma-Aldrich) and probed with anti-CRH(1:100, Signalway) or anti-NPW (1:100, orb482515, Biobyt) and anti-FoxO3a (1:100, Cat. No. #12829, Cell Signaling Technology) at 4℃ for 2 days. After washing 3 times with PBS, they were incubated with conjugated donkey anti-goat secondary antibody Alexa 594 red (Serotec,) or conjugated donkey anti-rabbit antibody Alexa 488 green (Serotec) at room temperature for 1 h. The images were acquired with a fluorescence microscope (X 20 objective lens).

### Statistical analysis

All experiments were performed in triplicates. The data significance was evaluated by SPSS 11.5 software. All values were presented as mean ± SD. Statistical significance among various groups was calculated by one-way ANOVA using post hoc multiple comparisons, when p<0.05 was considered statistically significant.

## Results

### Dexamethasone (DEX) decreased mRNA and protein expression of NPW but increased FoxO3a expression in PC12 and primary hypothalamic neuronal cell cultures

**Fig 1:**
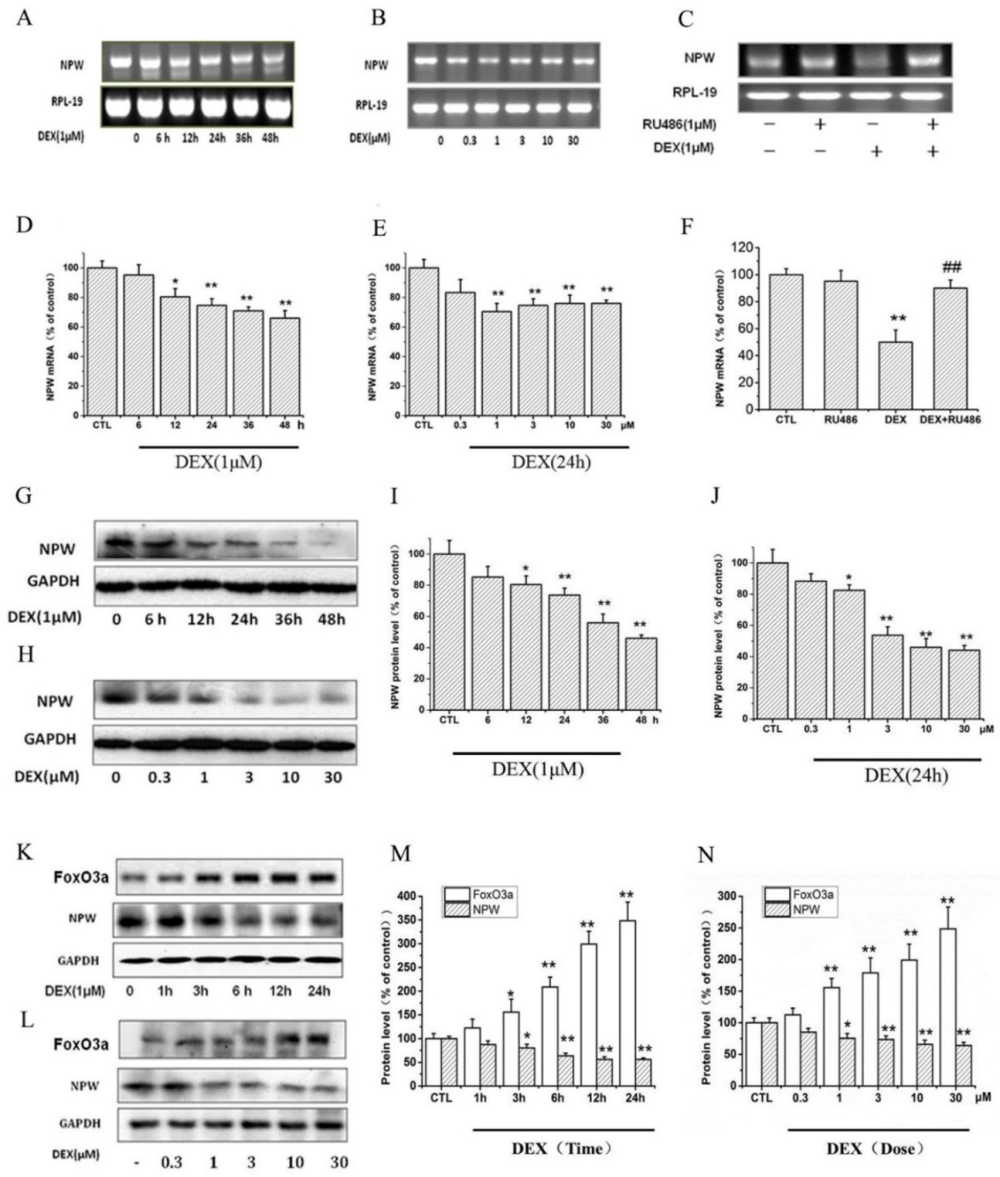
The effect of DEX in the mRNA and protein level of NPW and FoxO3a. The mRNA level of NPW in PC12 cell cultures was analyzed, (A) Time-course for treatment with 1μM DEX; (B) Dose-dependency after treatment for 24 h; (C) PC12 cells were pretreated with 1μM RU-486 for 1 h, prior stimulation with DEX, followed by 24 h treatment with 1μM DEX; (D, E, F) Densitometric analysis of the gel blot expressed as percentage of control. ^*^p<0.05, ^**^p<0.01 vs control group; ^##^*p*<0.01 versus DEX group. (G, H) The protein levels of NPW in PC12 cells were analyzed by western blotting and GAPDH were used as a loading control; (K, L) Representative images of western blots of time course and dose-dependency of DEX-induced increased expression of FoxO3a and inhibition of expression of NPW proteins in primary hypothalamic neurons. (I, J, M, N) Densitometric analysis of the immunoblots expressed as percentage of control. ^*^p<0.05, ^**^p<0.01 vs control group. The assay was repeated 3 times.

Dexamethasone (DEX) reduced the expression level of NPW mRNA in PC12 cells in a time-dependent manner (Fig. 1 A, D). 1 μM DEX decreased the expression level of NPW mRNA from 6 h and reached a maximal 30% reduction at 48 hours post-incubation (Fig. 1 A, D). Downregulation of NPW mRNA by 24 hours DEX treatment was also concentration-dependent (Fig. 1B, E) with the maximal 30% effect observed at 1μM. DEX-induced decrease of NPW mRNA expression in PC12 cells was significantly attenuated by pretreatment with 1 μM glucocorticoid type 2 (GR-2) receptor antagonist RU-486 (Fig 1. C, F), indicating that the effect was mediated by GR-2 receptors. Consistent with these findings, results from western blotting experiments also showed that DEX induced a significant reduction of NPW protein expression in PC12 cells in a time (Fig. 1 G, I) and concentration (Fig. 1 H, J) dependent manner. Treatment of the PC12 cells for 48 hours with 1 μM (Fig. 1G, I) or for 24 hours with 10 μM DEX (Fig. 1H, J), clearly indicated a maximal significant reduction of 50% expression of NPW protein. To further confirm this effect and correlate it to FoxO3a expression in target neurons, brain primary hypothalamic neurons were treated with DEX for different periods of time, and at different concentrations, and the protein levels of both FoxO3a and NPW were determined by western blotting. The results revealed that DEX increased the expression of FoxO3a in parallel to the decreased expression of NPW in a time (Fig. 1 K, M) and concentration dependent manner (Fig. 1 L, N).

### Dexamethasone (DEX) decreased Akt, FoxO3a phosphorylation and enhanced FoxO3a nuclear localization in PC12 cells

**Fig 2:**
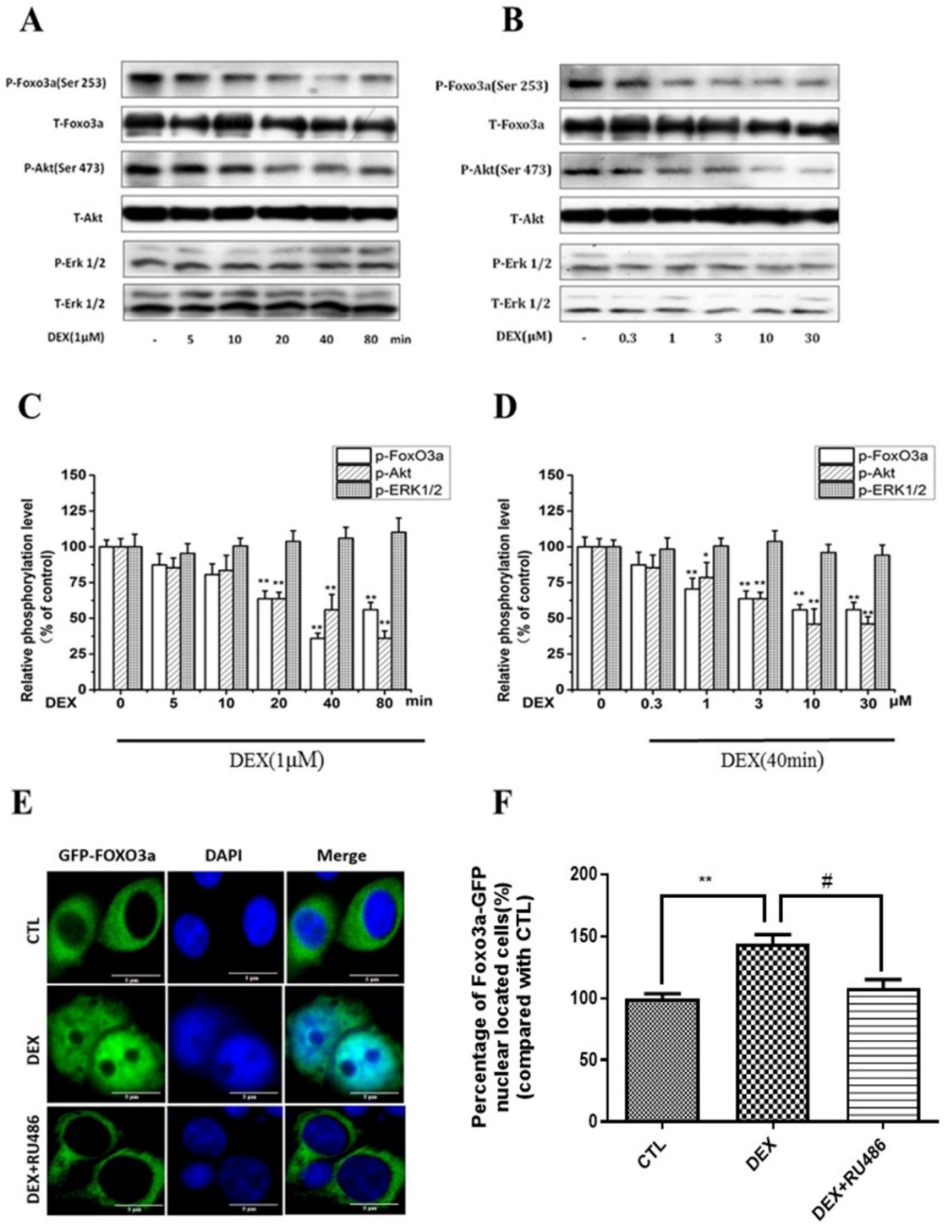
DEX treatment of PC12 cell cultures decreased Akt, FoxO3a, but not ERK1/2 phosphorylation. (A) Time course with 1µM DEX (B) Dose-dependency after 40 min treatment. Densitometry of the immunoblots (upper part-A, B) was expressed as a percentage of control in the middle part (C, D). Representative pictures of DEX effect on nuclear translocation of FoxO3a in PC12 cells (E, F). PC12 cells transfected with GFP-FoxO3a and cultured with either10% FBS DMEM, or treated with 1µM DEX, or co-treated with 1µM DEX and 1µM RU486 (E, F). Immunofluorescent staining indicating subcellular localization of FoxO3a was performed as described in Material and Methods. Results represent mean ± SD of three independent experiments. ^*^p< 0.05; ^**^p<0.01 versus control group;^ #^*p*<0.05 versus DEX group. The assay was repeated 3 times.

We have previously reported that corticosterone decreased the phosphorylation of FoxO3a and Akt in a concentration- and time-dependent manner in PC12 cells, in correlation to FoxO3a nuclear shuttling and cell apoptosis 31. Therefore, we sought to investigate the possible involvement of Akt and FoxO3a in DEX inhibitory effects on NPW expression. For this purpose, PC12 cells were treated with 1μM DEX for different periods of time, or different concentrations for 40 min. The simultaneous assessment of phosphorylated and non-phosphorylated Akt, FoxO3a and Erk 1/2 levels in the same cell culture samples was performed. The data indicated that 1μM DEX time-dependently, inhibited the phosphorylation of FoxO3a and Akt and reached maximal inhibition by 60% and 40% respectively in 80 min, with no effect on Erk 1/2 phosphorylation (Fig. 2 A, C). A concentration-dependent effect was also obtained after treatment of PC12 cultures with different concentrations of DEX for 40 min, with a maximal inhibition at 10 μM DEX (Fig. 2 B, D). Phosphorylation and dephosphorylation of FoxO3a are important for its function and subcellular localization 32. While phosphorylation of FoxO3a causes its cytoplasmic localization and its dysfunction and degradation, dephosphorylation enhances its nuclear localization (cytoplasmic-nuclear shuttling) and gene expression. To investigate the effect of DEX on the cytoplasmic-nuclear shuttling of FoxO3a, PC12 cells transfected with GFP-FoxO3a were cultured in 10% FBS DMEM only or treated with 1µM DEX or co-treated with 1µM DEX and 1µM RU 486. The subcellular localization of FoxO3a was determined by fluorescence microscopy. The results indicated that DEX induced FoxO3a translocation into the nucleus and that RU 486 reversed this effect (Fig. 2 E, F). Cumulatively, these results indicate that DEX reduced the phosphorylation of Akt and affected the subcellular localization and phosphorylation of FoxO3a.

### Overexpression or knockdown of FoxO3a decreased or increased the expression of NPW transcript

**Fig 3:**
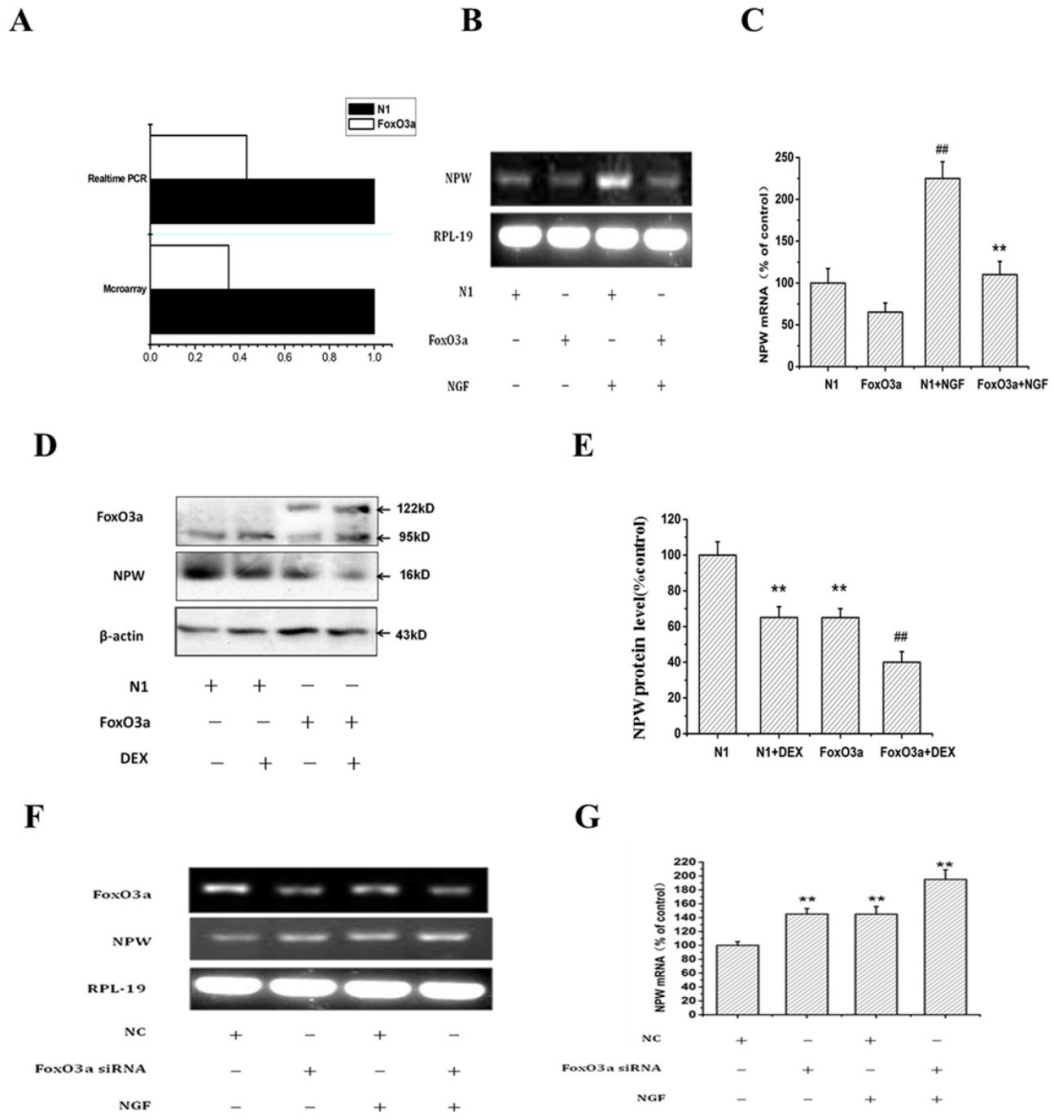
Effects of FoxO3a on the level of NPW in PC12 cells. (A) PC12 cells were transfected with GFP-FoxO3a or GFP-N1, and 36 h after transfection, gene expression profiles in the cells transfected with GFP-FoxO3a vs. GFP-N1 were investigated. Real-time PCR and microarray were carried out to determine the mRNA levels of NPW and the alteration of gene expression profile; the fold change of NPW in the FoxO3a-overexpressing cells over control was obtained by setting the value from the control to one. (B-E) PC12 cells were electro-transfected with control plasmid GFP-N1 or GFP-FoxO3a, and 36 h after transfection, cells were treated with or without 50ng/ml NGF (B,C), or with or without dexamethasone(D,E), and the effect of FoxO3a overexpression on the expression of NPW was studied by RT-PCR or western blotting. (F, G) PC12 Cells were transfected with FoxO3a specific siRNA and the scrambled siRNA (NC), 36 hours after transfection, cells were treated with or without 50 ng/mL NGF for 24 h, the mRNA level was detected by RT-PCR. ^**^p<0.01 versus GFP-N1 group, ^##^*p*<0.01 versus GFP-FoxO3a group. The assay was repeated 3 times.

To understand the relationship between FoxO3a and NPW, we performed the microarray assay using individual Nimble Gen-tiled microarray chip containing 385,0000 probes tiled at a resolution of 1 probe per 100-180 bp, to identify genomic‐binding sites of FoxO3a in the transfected PC12 cells. Compared to randomly selected regions of the genome, promoter regions identified of NPW by ACME (Algorithm for Capturing Microarray Enrichment) reported 3,000 probes to be enriched at p <0.0001, reliably representing sites of FoxO3a occupancy. Among them, the level of NPW in FoxO3a-transfected PC12 cells was 2.7 times lower than that in cells transfected with control plasmid N1. This finding was validated by qRT-PCR (Fig. 3 A).

To further investigate the effect of FoxO3a on NPW expression, PC12 cells were electro-transfected with control plasmid GFP-N1 or GFP-FoxO3a and treated with or without DEX, NGF (was used to stimulate the expression of NPW), and the effect of FoxO3a on the expression of NPW mRNA and protein was studied by RT-PCR and western blotting, respectively. RT-PCR results revealed that FoxO3a overexpression (under basal conditions or induced by NGF) inhibited the mRNA expression (transcription) of NPW by 40% in hypothalamic neuronal cultures (Fig. 3 B, C). Western blotting analysis showed DEX decreased the expression of NPW, and FoxO3a enhanced the effect of DEX in inhibiting NPW expression (Fig. 3 D, E), indicating that FoxO3a has an inhibitory regulatory effect on the expression of NPW in both non transfected and FoxO3a overexpressing cells. To further address the inhibitory role of FoxO3a on NPW transcription, we knocked down the expression of FoxO3a in wild type PC12 cells by a specific siRNA. Results indicated that the mRNA level of NPW was elevated in cells transfected with FoxO3a siRNA under basal conditions or induced by NGF (Fig. 3 F, G).

### FoxO3a bound to NPW gene promoter and inhibited its transcriptional activity

**Fig 4:**
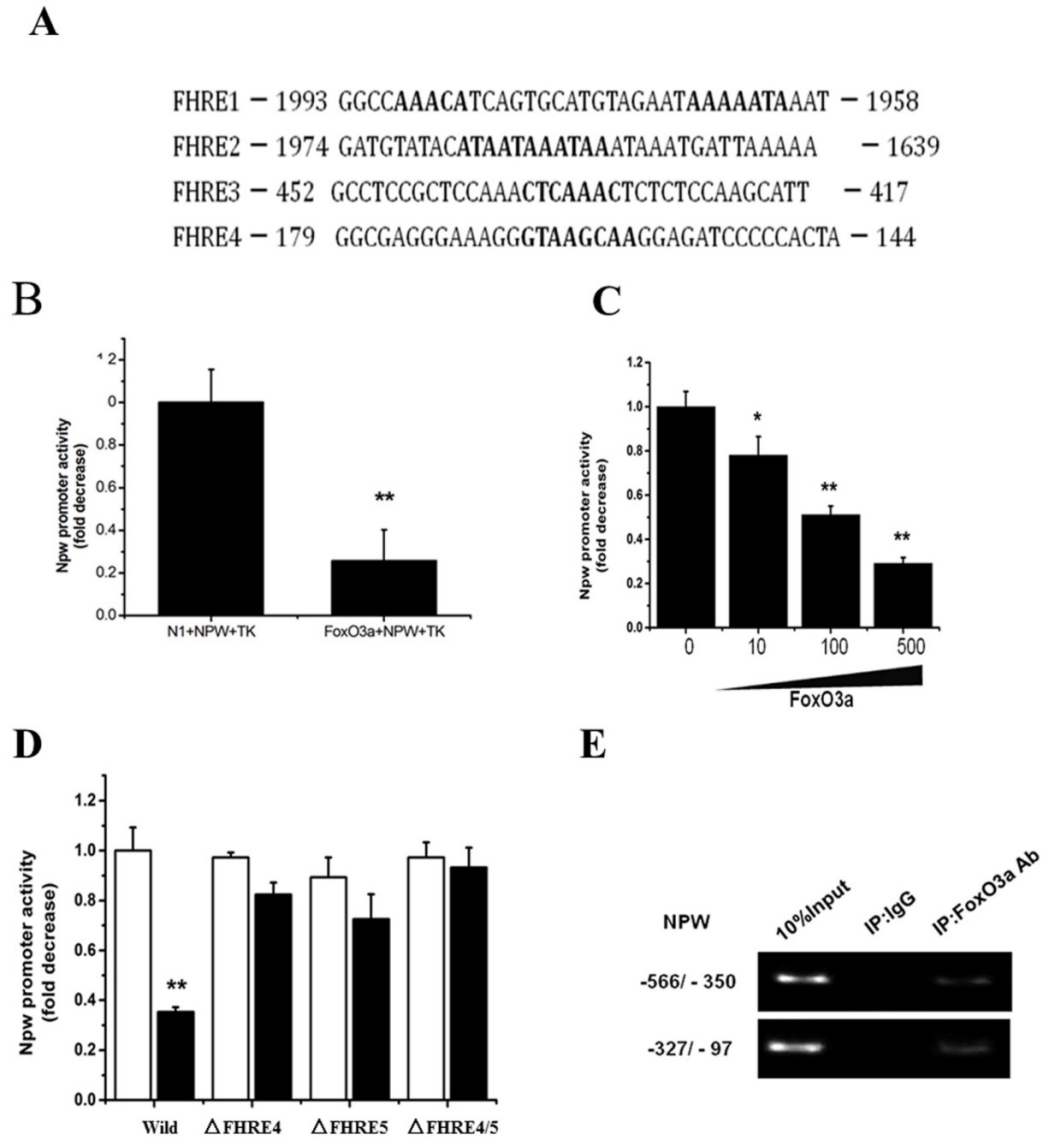
FoxO3a binds to the NPW promoter and inhibits NPW transcriptional activity. (A) The potential FoxO3a-binding sites (Forkhead responsive elements, FHREs) in the 2-kb promoter region of the human NPW gene; (B) Effects of FoxO3a on the transcription activity of NPW in PC12 cells. NPW promoter and FoxO3a were co-transfected into PC12 cells; the luciferase activity was measured from cell lysate; (C) Expression of FoxO3a (10 ng, 100 ng and 500 ng) decreased NPW promoter activity in PC12 cells. ^*^p< 0.05; ^**^p< 0.01 versus mock-transfected control; (D) FoxO3a did not affected the NPW promoter activity when FHRE4, FHRE5 or both were deleted (ΔFHRE4, ΔFHRE5); ^*^p<0.01 versus wild-type NPW-luc transfected control ('Wild'); (E) Chromatin immunoprecipitation assay showing endogenous FoxO3a binding to the NPW promoter region (-437/-431 and -166/-159); the PCR product of the NPW promoter region amplification (nucleotides -350/-566 and -97/-327) containing FHRE4 and FHRE5. The assay was repeated 3 times.

The above-mentioned results (Fig. 3) propose that NPW gene may be a new downstream target of FoxO3a. To further validate this possibility, we searched the putative FoxO3a-consensual binding sites in NPW promoter region, -2-kb distance from the transcription start site, using common bioinformatics software tools (http://genome.ucsc.edu/). We identified four potential Forkhead-binding sites (FHRE) at positions -158, -431, -1751, and -1965 in NPW gene promoter. These findings suggest that NPW gene is a novel target of FoxO3a transcription factor (Fig. 4A). To confirm this hypothesis, a part of NPW gene promoter (nucleotides -900 to +100) was cloned and transferred into a reporter gene plasmid. Dual-Luciferase assay with this reporter plasmid showed that FoxO3a significantly inhibited the NPW gene promoter activity by 60 % (Fig.4 B), an effect that was dependent of FoxO3a concentration, upon co-transfection of NPW and FoxO3a genes in the same PC12 cell cultures (Fig.4 C). As presented in Fig. 4A, there are several potential FoxO3a binding sites in the NPW gene promoter and therefore we questioned which binding site(s) is essential for the inhibitory effect of FoxO3a on NPW promoter activity. To clarify this issue, we prepared different reporter plasmids with NPW gene promoter in which, each binding site of FoxO3a was mutated. The results indicated that the deletion mutants of FoxO3a binding sites (FHRE4 and FHRE5), created by removal of region -437/-431 and -166/-159, respectively, are obligatory for FoxO3a-induced NPW promoter activity (Fig.4 D). Consistent with these findings, ChIP assay also revealed that endogenous FoxO3a binds to the promoter of NPW in the region between nucleotides -431 and -158, upstream of the NPW coding sequence, which includes the FHRE4 and FHRE5 binding sites (Fig. 4 E). Cumulatively, these studies support the concept that NPW gene is a new downstream target of FoxO3a transcription factor in neurons.

### Glucocorticoid regulation of FoxO3a and NPW mRNA and protein expression in the rat hypothalamus

**Fig 5:**
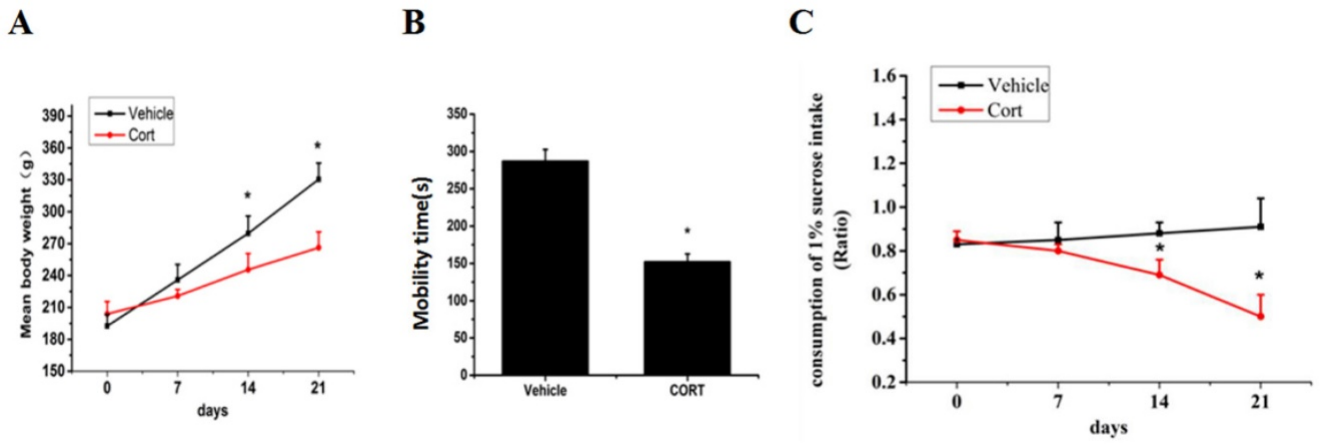
Chronic corticosterone (CORT) administration reduced animals' body weight and sucrose consumption and caused stress depression-like behavior. (A) The mean body weights of the rats in each group during the 21-day stress phase of the experiment. (B) Mean percent of time spent mobile during the forced swim test. (C) Consumption of sucrose up to 21 days. The body weight and the consumption of 1% sucrose intake in vehicle groups and CORT groups were tested once weekly. ^*^ *p*<0.05 compared with vehicle group rats. The assay was repeated for 3 times.

**Fig 6:**
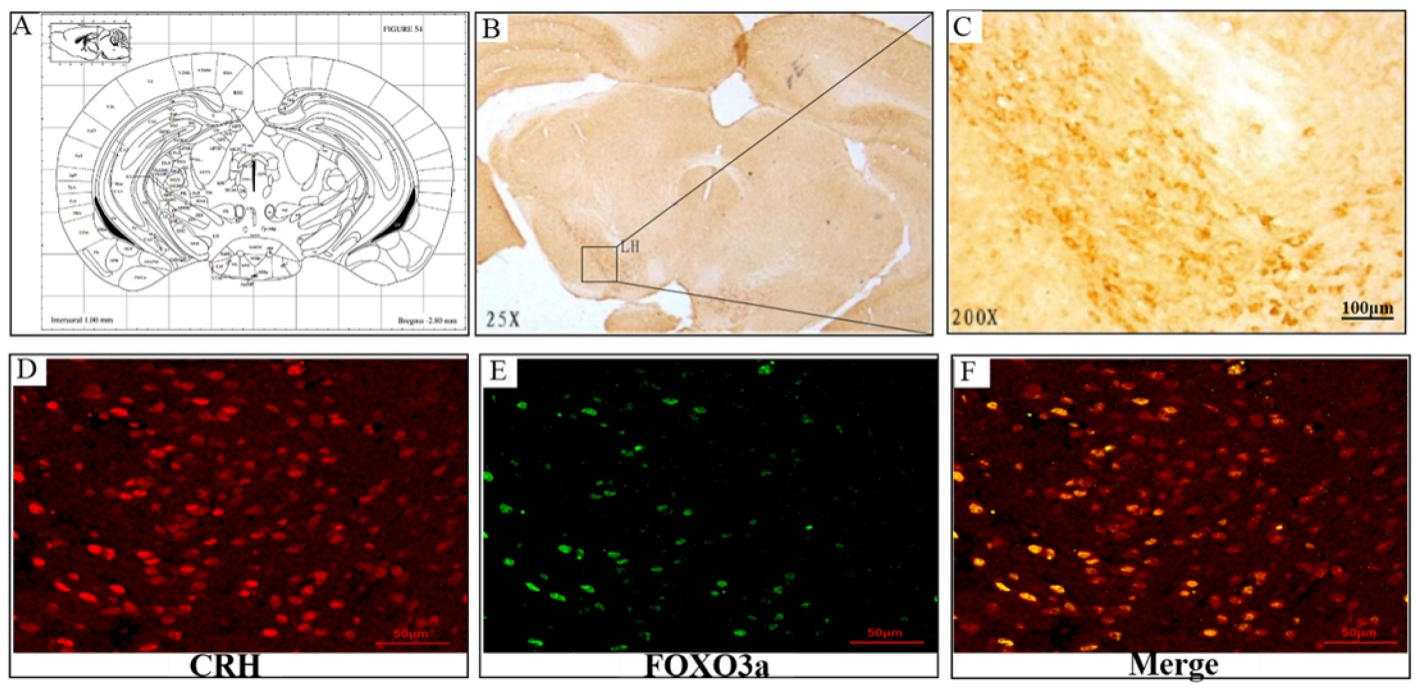
Representative sections of the brain hypothalamus of male Sprague-Dawley rats, stained immunocytochemically for FoxO3a (position: Interaural 1.00 mm, Bregma -2.80 mm). (A) FoxO3a-immunoreactive neurons are seen in the hypothalamic area, (B) 25x magnification, (C) 200X magnification of lateral hypothalamic area. (D-F) FoxO3a expression in CRH-positive neuron in the hypothalamic area (Co-localization of CRH and FoxO3a).

**Fig 7:**
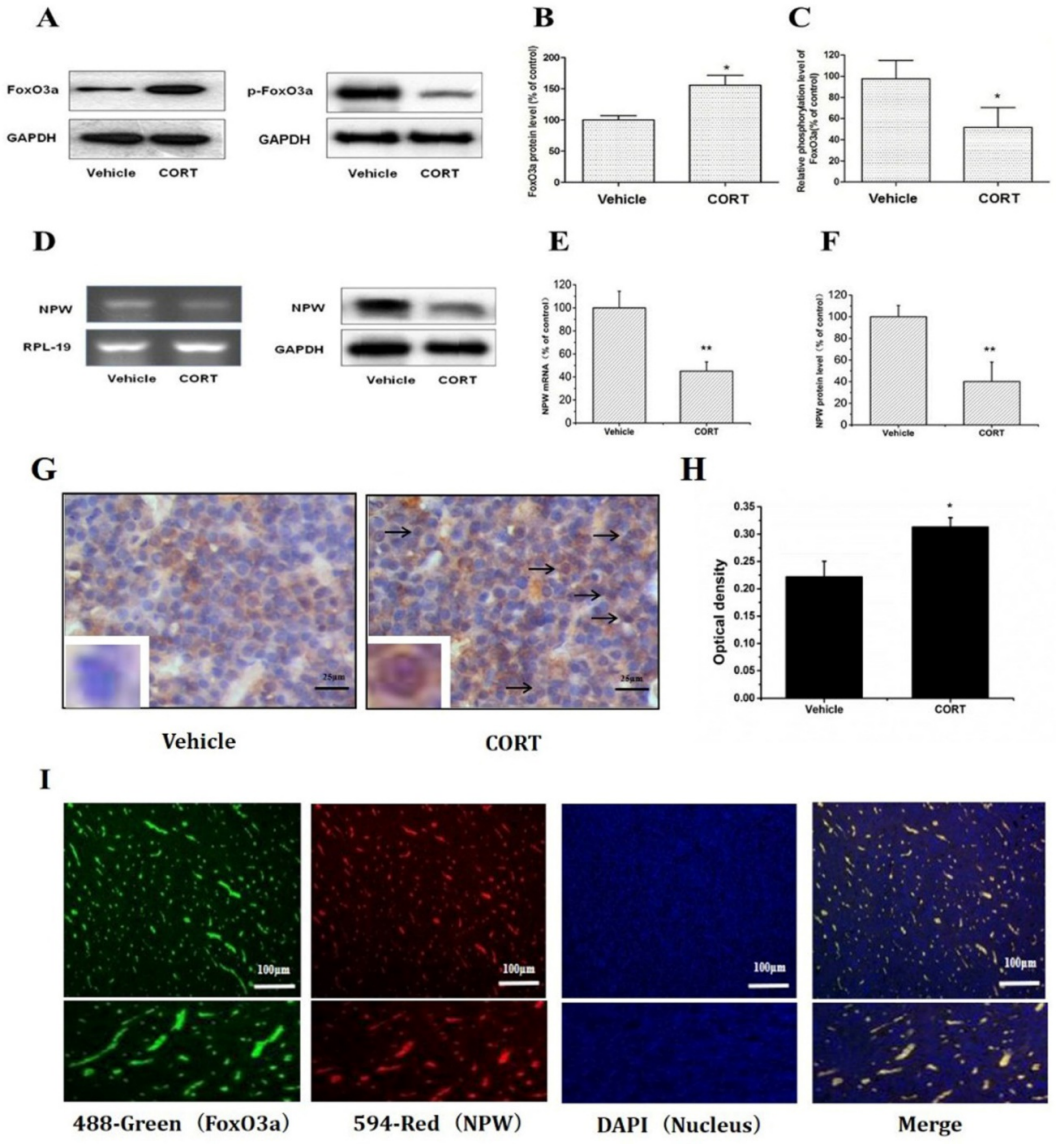
FoxO3a and NPW protein and mRNA levels in the hypothalamus of male Sprague-Dawley (SD) rats receiving corticosterone (CORT, 40 mg/ml/kg) or saline for 21 days (n =15 in each group); (A-F) Administration of corticosterone increased FoxO3a protein level while decreased the phosphorylation of FoxO3a, NPW mRNA and its protein expression in the hypothalamus of the rat,^ *^ *p*<0.05 compared with vehicle group rats; (G) The area density of FoxO3a expression in CORT group was significantly higher than that in the control group. The optical density in the CORT group was also significantly higher than that in the control group(H). ^*^ *p*<0.05 compared with vehicle group rats; (I) Colocalization of FoxO3a and NPW in hypothalamus of rat. Dual immunofluorescence photomicrographs of FoxO3a (green) and NPW (red)-immunoreactive neurons in the hypothalamus, NPW-immunoreactive fibers (red) are in close apposition with FoxO3a-immunoreactive neurons (green) in the the hypothalamus. The assay was repeated 3 times.

Chronic corticosterone (CORT) administration in rodents is used in pharmacological animal models of stress-induced depression 29, 33, 34. We adopted this model to study mRNA and protein expression of FoxO3a and NPW in the brain hypothalamus of corticosterone treated rats. Our results showed that CORT administration significantly reduced the body weight, the sucrose intake and mobility time during the forced swim test (Fig. 5). These findings are consistent with the CORT-induced depression model in rat as displaying a depressive-like behavior. Immunohistochemically verification of FoxO3a protein localization in the hypothalamus of control animals, abundance of FoxO3a-immunoreactive neurons in the hypothalamus and FoxO3a expression in CRH-positive neuron (Fig. 6). In the next experiments, RT-PCR and western blotting methods were used to measure the expression levels of NPW mRNA and protein level, while western blotting method was employed to detect FoxO3a protein levels and its phosphorylation in the rat hypothalamus. CORT treatment increased the expression level of FoxO3a protein (Fig. 7A, B) while decreased the phosphorylation of FoxO3a (Fig 7 A, C). As found in neuronal cultures, CORT treatment reduced the mRNA and protein level of NPW by 58% in the rat model (Fig. 7D, E, F). Assessment of FoxO3a expression in the hypothalamus by immunocytochemistry (Fig.7 G, H), revealed that CORT administration induced a significant increase of FoxO3a expression and caused nuclear translocation of FoxO3a compared with the control group. Double immunofluorescence labeling revealed that FoxO3a and NPW are colocalized in hypothalamus (Fig. 7 I). FoxO3a-immunoreactive neurons were observed in the lateral hypothalamic area. Neurons expressing NPW were also detected. These neurons were characterized by different morphology and size (20-30 microns diameter). The immunofluorescence photomicrographs of the neural interaction between NPW (red) and FoxO3a (green)-labeled neurons indicated that NPW-immunoreactivity expressing fibers were in close apposition with FoxO3a-immunoreactive neurons (Fig. 7 I). Cumulatively, these in vivo studies of chronic administration of corticosterone indicate an indirect correlation between the marked increase in FoxO3a levels in hypothalamus and concomitant decrease of NPW levels and FoxO3a phosphorylation levels.

## Discussion

In this study, we explored the effect of dexamethasone (DEX), a typical synthetic corticosteroid, on the mRNA and protein levels of NPW in PC12 cells and primary cultured hypothalamic neurons. We have previously reported that corticosterone decreased the phosphorylation of Akt and its downstream targets FoxO3a by the PI3K pathway in a PC12 cell culture neuronal model 31. We also uncover the important role of PI3K/Akt pathway on DEX-induced expression, phosphorylation and subcellular localization of FoxO3a in relation to corticosterone-induced cell death in the same neuronal model 26. In order to study how DEX regulates NPW expression, we used the RT-PCR and western blotting approaches to determine the optimal kinetics and dose-dependency of DEX treatment. We found DEX stimulated the expression of FoxO3a while inhibited the expression of NPW in a dose-and time-dependent manner, in both PC12 cells and primary cultured hypothalamic neurons. While glucocorticoid receptor antagonist RU 486, significantly decreased the inhibitory effect of DEX on NPW mRNA and protein levels (Fig. 1). Moreover, its effect was related to the decrease in Akt phosphorylation and the significant change in the subcellular localization and phosphorylation of FoxO3a (Fig. 2).

Consistent with these results, we also found that overexpression of FoxO3a in PC12 cells reduced the expression of NPW and attenuated the stimulatory effect of NGF or DEX on NPW expression (Fig 3A-E), while knocking down FoxO3a significantly increased the mRNA level of NPW (Fig. 3F-G). We predicted multiple FoxO3a binding sites in the regulatory region of NPW gene promoter and the results from ChIP assay strongly indicated that FoxO3a could directly bind to the promoter region of NPW (Fig. 4 E). NPW promoter (nucleotides -900 to +100) was cloned and constructed into a gene plasmid, and the dual-luciferase reporter assay indicated that FoxO3a inhibited the transcriptional activity of NPW in PC12 cells (Fig. 4B-C). Identify the binding site(s) of NPW promoter responding to FoxO3a, we constructed NPW promoter's deletion mutants and used them in luciferase reporter assay. FoxO3a failed to down-regulate NPW promoter activity in deletion mutant FHRE4 and FHRE5, which were generated by removing region -437/-431 and -166/-159 respectively. Moreover, luciferase reporter assay using deletion mutants carrying mutation of each FoxO3a-binding site demonstrated that these binding sites are indeed required for the NPW gene promoter to respond to FoxO3a (Fig. 4D).Taken together, these results indicate that NPW gene is a direct target of FoxO3a, which suppresses the transcription of NPW protein in PC12 and primary cultured hypothalamic neurons models.

The present findings are reinforced by studies indicating that FoxO3a negatively regulates: i. NGF-induced neuronal differentiation in PC12 cells, by binding to the promoter and decreasing the transcription of neurochondrin gene 18; ii. IGF-1 induced neuroprotection towards β-amyloid toxicity in neuroblastoma SH-SY5Y cell cultures, by binding to PUMA (p53 upregulated modulator of apoptosis) promoter, leading to decreased PUMA expression 35. However, there are also studies showing that FoxO3a positively regulates: i. NGF withdrawal-induced cell death by binding to BIM (a BH3-only member of the BCL-2 family) promoter and activating its transcription in superior cervical ganglia sympathetic neurons 36; ii. Poly (ADP-ribose) polymerase-1 (PARP1) -induced mitochondrial dysfunction and neuronal cell death by enhancing association of FoxO3A with Bcl2/adenovirus E1B 19 kDa interacting protein (Bnip3- a pro-apoptotic member of the BH3-only subfamily of Bcl-2 proteins) promoter, increasing its transcription and elevating its mitochondrial immunoreactivity in primary mouse brain cortical neuronal cultures 37; iii. Leptin regulation of brain-adipose feedback axis in energy metabolism by FoxO3a binding to cystathionine-β-synthase (CBS) promoter and then enhanced CBS protein expression in primary neonatal rat thalamus neuronal cultures and the hypothalamus of Sprague-Dawley rats 38. It is plausible that the opposite transcriptional effects of FoxO3a on the target gene promoters is due to differences in the cell systems used, i.e. the dopaminergic PC12 and SH-SY5Y neuronal cultures versus primary cultures of superior cervical ganglia sympathetic neurons, brain cortical and thalamic neuronal cultures. Alternatively, this may be due to the complex mechanisms of regulation of target gene by multiple transcription factors in addition to FoxO3a, which remain to be fully identified. Here, we show that the FoxO3a, whose activity is known to be repressed by the PI3K/Akt kinase-signaling cascade, is an important transcriptional negative regulator of the gene encoding the NPW protein. Since FoxO3a regulates cell survival in response to DNA damage, oxidative stress and caloric restriction, it is tempting to propose that NPW, being regulated by FoxO3a, is also involved in the signaling regulatory cassette of these physiological and pathological functions.

Chronic corticosterone administration to SD rats caused a depressive-like state (Fig. 5) and induced a marked increase in FoxO3a levels while decreased the expression of NPW protein in the hypothalamus (Fig. 7). Chronic stress induces altered energy metabolism and plays important roles in the etiology of depression, in which the glucocorticoid negative feedback is disrupted due to unbalanced glucocorticoid receptor functions. The mechanisms underlying this dysregulation remain elusive. Present data may suggest that FoxO3a and NPW protein levels and activity are part of this mechanism and that dysfunction of FoxO3a and NPW protein expression or activity may be involved in the pathogenesis of stress-induced depression.

## Conclusion

In summary, we detected dexamethasone-induced inhibition of FoxO3a-mediated NPW expression in neuronal cultures and rat hypothalamus. We also demonstrated that glucocorticoid administration reduced the phosphorylation of FoxO3a in hypothalamus, a process correlated with the suppression of NPW protein expression. Altogether, our findings demonstrate that the NPW, a novel peptide hormone and FoxO3a transcription factor, may represent novel targets for the prevention and/or treatment of chronic stress-induced psychiatric disorders, including depression.
